# Supplementary material for: Prostate Cancer Screening with PSA: Ten Years’ Experience of Population Based Early Prostate Cancer Detection Programme in Lithuania
Source: J Clin Med. 2020 Nov 26;9(12):3826. doi: 10.3390/jcm9123826 (PMC7760278; doi:10.3390/jcm9123826)
Supplement: Supplementary file 1 [file jcm-09-03826-s001.pdf]

Supplementary

Table S1. Main performance indicators of Early Prostate Cancer Detection Programme (EPCDP) in Lithuania in the first seven screening rounds between 2006 and 2015 for men, aged 45-49 years.

.	**Calendar year (screening round)**						
	**2006 (R1)**	**2007 (R2)**	**2008 (R3)**	**2009 (R4)**	**2010-2011 (R5)**	**2012-2013 (R6)**	**2014-2015 (R7)**
Individuals screened (45–49-year old)	1872	2075	2415	1873	2563	2134	1687
PSA results							
PSA <3 ng/mL (%)	1735 (92.7)	1928 (92.9)	2255 (93.4)	1755 (93.7)	2390 (93.3)	1993 (93.4)	1573 (93.2)
PSA ≥3 ng/mL (%)	137 (7.3)	147 (7.1)	160 (6.6)	118 (6.3)	173 (6.7)	141 (6.6)	114 (6.8)
Biopsy							
Number of biopsies (% of PSA positive)	23 (16.8)	53 (36.1)	43 (26.9)	54 (45.8)	81 (46.8)	54 (38.3)	62 (54.4)
Prostate cancer (% of biopsy)	5 (21.7)	19 (35.8)	17 (39.5)	22 (40.7)	20 (24.7)	22 (40.7)	26 (41.9)
% prostate cancer of test-positive	3.6	12.9	10.6	18.6	11.6	15.6	22.8
% prostate cancer of screened persons	0.3	0.9	0.7	1.2	0.8	1.0	1.5

PSA – prostate specific antigen; R - round.
